# Using mind control to modify cue-reactivity in AUD: the impact of mindfulness-based relapse prevention on real-time fMRI neurofeedback to modify cue-reactivity in alcohol use disorder: a randomized controlled trial

**DOI:** 10.1186/s12888-020-02717-7

**Published:** 2020-06-16

**Authors:** Franziska Weiss, Acelya Aslan, Jingying Zhang, Martin Fungisai Gerchen, Falk Kiefer, Peter Kirsch

**Affiliations:** 1 Department of Clinical Psychology, Central Institute of Mental Health (ZI), Heidelberg University/Medical Faculty Mannheim, 68159 Mannheim, Germany; 2 Department of Addiction Behavior and Addiction Medicine, Central Institute of Mental Health, Heidelberg University/Medical Faculty Mannheim, Mannheim, Germany; 3 Department of Psychology, Heidelberg University, Heidelberg, Germany; 4 Bernstein Center for Computational Neuroscience Heidelberg/Mannheim, Mannheim, Germany

**Keywords:** rtfMRI neurofeedback, Ventral striatum, Alcohol use disorder, Cue-reactivity, Alcohol dependence, Addiction, Mindfulness-based relapse prevention, Mindfulness, Mindfulness-based treatment

## Abstract

**Background:**

Alcohol Use Disorder is a severe mental disorder affecting the individuals concerned, their family and friends and society as a whole. Despite its high prevalence, novel treatment options remain rather limited. Two innovative interventions used for treating severe disorders are the use of real-time functional magnetic resonance imaging neurofeedback that targets brain regions related to the disorder, and mindfulness-based treatments. In the context of the TRR SFB 265 C04 “Mindfulness-based relapse prevention as an addition to rtfMRI NFB intervention for patients with Alcohol Use Disorder (MiND)” study, both interventions will be combined to a state-of-the art intervention that will use mindfulness-based relapse prevention to improve the efficacy of a real-time neurofeedback intervention targeting the ventral striatum, which is a brain region centrally involved in cue-reactivity to alcohol-related stimuli.

**Methods/design:**

After inclusion, *N* = 88 patients will be randomly assigned to one of four groups. Two of those groups will receive mindfulness-based relapse prevention. All groups will receive two fMRI sessions and three real-time neurofeedback sessions in a double-blind manner and will regulate either the ventral striatum or the auditory cortex as a control region. Two groups will additionally receive five sessions of mindfulness-based relapse prevention prior to the neurofeedback intervention. After the last fMRI session, the participants will be followed-up monthly for a period of 3 months for an assessment of the relapse rate and clinical effects of the intervention.

**Discussion:**

The results of this study will give further insights into the efficacy of real-time functional magnetic resonance imaging neurofeedback interventions for the treatment of Alcohol Use Disorder. Additionally, the study will provide further insight on neurobiological changes in the brain caused by the neurofeedback intervention as well as by the mindfulness-based relapse prevention. The outcome might be useful to develop new treatment approaches targeting mechanisms of Alcohol Use Disorder with the goal to reduce relapse rates after discharge from the hospital.

**Trial registration:**

This trial is pre-registered at clinicaltrials.gov (trial identifier: NCT04366505; WHO Universal Trial Number (UTN): U1111–1250-2964). Registered 30 March 2020, published 29 April 2020.

## Background

Alcohol Use Disorder (AUD) is a severe and widespread disease that affects millions of individuals worldwide (World Health Organization (WHO) [54]; Shield et al. [48]). It is characterized by an uncontrollable urge to consume alcohol despite severe psychological or physical health consequences, a loss of social connections, a lack of interests in former joyful activities and an increased tolerance [1]. The consequences not only concern the affected individual but also the social circle (e.g. family, friends, colleagues) and society as a whole [36]. Often, affected individuals are in need of extensive health care and cannot resume their work for many years, which generates high costs for the health care system [3].

Despite the consequences described above, treatment options for AUD are still limited in their efficacy and reach. Only 19.8% of patients with lifetime AUD are ever treated [27] and 45–75% of those relapse 1 year after treatment [6, 30]. To improve this deficient situation, novel treatment options are needed. Optimally, such treatment options would involve the individual patient and support self-efficacy while addressing specific disease-related processes.

A psychologically relevant neurobiological process that is a promising target for such an intervention is cue-reactivity. Cue-reactivity, describing the phenomenon of an elevated response to alcohol-related stimuli, is a frequently disrupted system in AUD patients. Neurobiologically, the elevated response during the presentation of the alcohol related stimuli can be observed in regions such as the anterior cingulate cortex (ACC) and ventral striatum (VS) [46]. Once the brain has habituated to this cue-reactivity, AUD is sustained by the underactivity of prefrontal control regions accompanied by an increased reactivity of the VS [43]. Dysfunctioning of the prefrontal cortical systems [26] including ventromedial prefrontal cortex (vmPFC) [4] and dorsolateral prefrontal cortex (dlPFC) [42] which are regions related to executive functioning moderate the alcohol-seeking motivation, and elicited activation of VS drives alcohol-seeking behavior. This circle maintains repeated alcohol use and relapse after treatment [40, 43, 46].

One of the most applied neuroscientific methods for the investigation of disturbed brain processes in mental disorders is functional magnetic resonance imaging (fMRI), which has provided enormous insights over the last decade. With real-time fMRI neurofeedback (rtfMRI NFB) it is now becoming possible to use fMRI also as an intervention technology. During rtfMRI NFB, the participant is lying in the magnetic resonance imaging (MRI) scanner and the activation of a brain region is measured. A feedback value representing activity of the target region is shown to the participant, while he or she is instructed to find a strategy to regulate the value in a specific direction. So far, rtfMRI NFB has been initially applied in a variety of clinical populations e.g. schizophrenia [13], autism spectrum disorders [10], major depressive disorder [56] and borderline personality disorder [44]. In the field of substance use disorders first studies have used it in nicotine addiction [38], as well as in AUD [14, 35].

While few rtfMRI NFB AUD studies exist, one study has shown that the method has the ability to decrease craving [35]. Furthermore, it was found in non-treatment seeking heavy social drinkers that VS activity can be decreased by means of rtfMRI NFB, which was accompanied by an increase of activity in the right inferior frontal gyrus [39] which is in line with the model of Naqvi & Morgenstern [43].

However, no clinical rtfMRI NFB study has experimentally tested how specific instructions or prior training influence the NFB effect. For example, in our ongoing rtfMRI NFB AUD study [25] patients are not given any instructions about cognitive strategies that might help them control their brain signal prior to the NFB intervention. Nonetheless, studies with healthy participants imply that given said instructions might enhance neurofeedback-based learning [11, 16].

One promising training strategy for rtfMRI in AUD might be mindfulness-based relapse prevention. Mindfulness, which originates in the Pali word “sati” and means being aware, attentive and remembering [5], is defined as “paying attention in a particular way: on purpose, in the present moment, and non-judgmentally” ([32], p. 4). Studies show that mindfulness has many evidence-based benefits, e.g. on the interpersonal, intrapersonal and affective level [2, 15, 33] and might be a useful addition to the therapy of severe disorders [12, 24]. Compared to standard relapse prevention and treatment as usual (TAU), patients receiving the mindfulness-based relapse prevention (MBRP) program showed a significantly decreased risk of relapse to substance use [8, 57].

In order to test whether a prior mindfulness-based training can enhance the therapeutic efficacy that helps the patients regain behavioral control over their alcohol use and learn neural self-regulation with the help of rtfMRI NFB, we therefore combine it here with a mindfulness-based training for addictive behaviors. In the collaborative research center TRR SFB 265 [29] C04 “Mindfulness-based relapse prevention as an addition to rtfMRI NFB intervention for patients with Alcohol Use Disorder (MiND)” study we will assess the influence of MBT on rtfMRI NFB performance and the clinical outcome of the NFB intervention on clinical AUD patients. Insights about potential additional modifiability of VS activity evoked by structured MBRP will be gained in addition to further information about NFB as a state-of-the-art treatment.

## Method

### Hypotheses

We will assess whether intervention success is associated with specific neural correlates and behavioral predictors of variable domains. A prior study has shown that patients who, preceding the intervention, are most responsive to VS cue-reactivity benefit most from the treatment [37]. Therefore, we assume that our study will show similar effects by discriminating between high- and low-responders being premised on their baseline VS cue-reactivity. We expect that the group receiving the combination treatment (real rtfMRI and MBRP) will profit most in terms of the outcome. Considering that relapse will not be defined as an exclusion criterion, we assume a decreased amount of heavy drinking days and a decreased sum of alcohol consumption. Additionally, it is assumed that the number of days until relapse will increase. In contrast to the TAU and sham NFB group, we expect that the other two groups will gain more of an advantage. As a secondary effect, we expect participants with a lower score on alcohol related questionnaires to show a superior long-term outcome in terms of a higher number of non-drinking days.

### Sample size, group assignment and recruitment

**Fig. 1:**
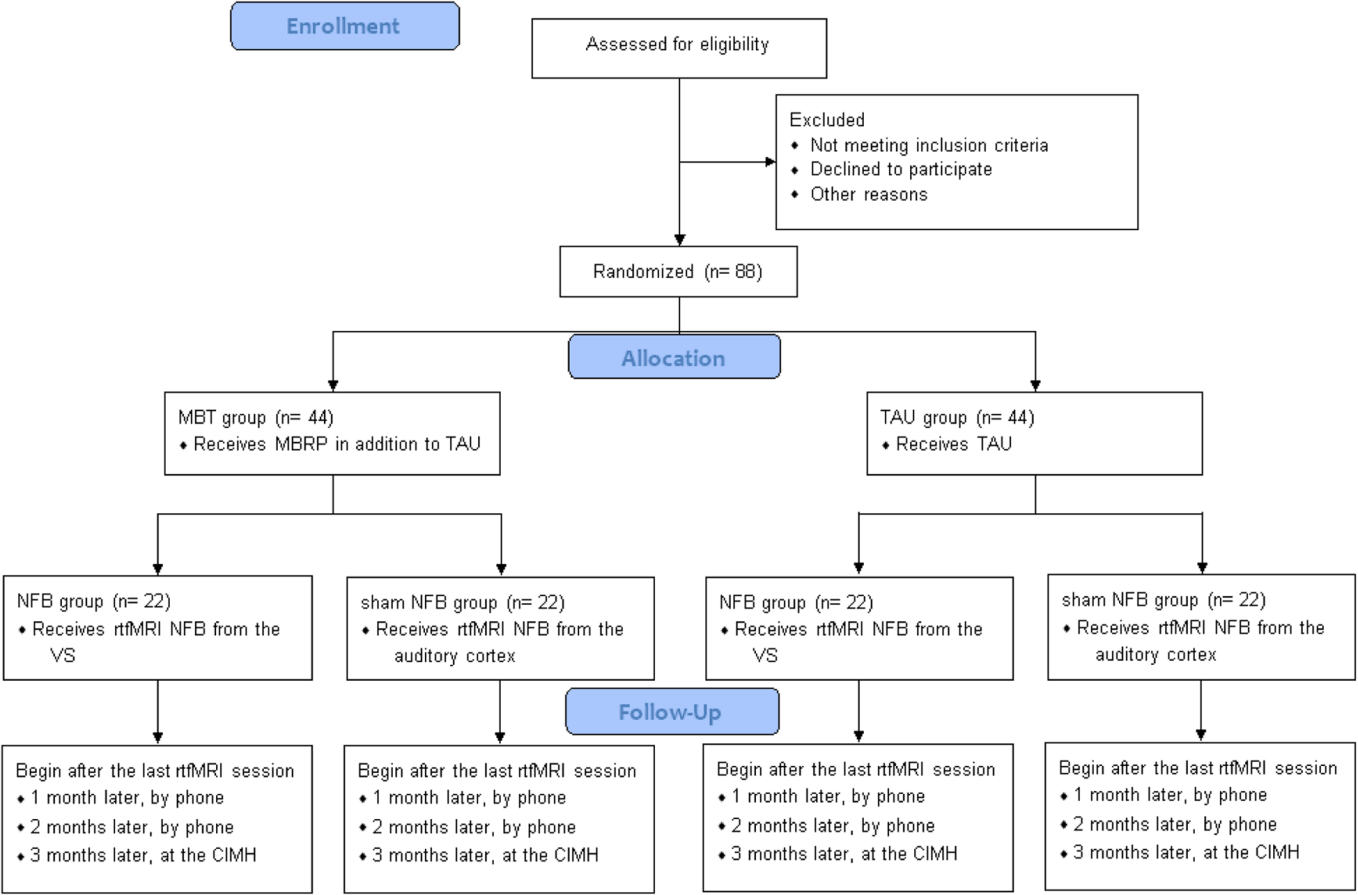
Flow chart. Flow diagram of the study procedure. *N* = 88 AUD participants will be enrolled in the study. They will randomly be assigned to one of four groups, which differ in terms of MBRP and NFB. All groups will be followed-up monthly for 3 months

In order to investigate a possible increased ability of our participants to deliberately reduce VS cue-reactivity through the help of MBRP, we will include *N* = 88 participants. These participants will be recruited from inpatients and outpatients of the clinics of the Department of Addiction Behavior and Addiction Medicine at the Central Institute of Mental Health (CIMH) Mannheim, Germany. After inclusion, they will be randomly assigned to four interventional groups (Fig. 1). While all groups will receive rtfMRI NFB from different brain regions, not all groups will be assigned to take part in the mindfulness-based treatment. Two groups will receive treatment as usual (TAU), whereas the other two groups receive MBRP. These two groups (TAU and MBT) will further each be divided in two subgroups, who receive either rtfMRI NFB from the VS with the instruction to down-regulate it (experimental group) or they receive feedback from the control region which is the auditory cortex (control group). The latter area was chosen since it is unrelated to cue-reactivity.

Participants assigned to the MBT groups will receive five sessions of MBRP in addition to TAU. In the first MBRP session, the patients receive an introduction to mindfulness. Furthermore, patients are taught to observe their thoughts and emotions with an interest but without judging. These sessions include the SOBER-Breathing training [7] which is a form of therapeutic direction using elements of meditation to reduce craving. In the last session, all relevant topics are repeated. Additionally, all participants in the MBRP group will be instructed to practice the shown mindfulness techniques for at least 15 min per day. After completion, the participants will receive the rtfMRI NFB intervention.

The allocation of the NFB technique (target region or control region) is double-blind. Since it is not possible to blind the patients on their additional treatment in form of MBRP, only the staff conducting the MRI scanning session will not be informed about the received treatment (MBRP or TAU) of the participant, which makes this part of the study single-blind.

### Participant eligibility, inclusion and exclusion criteria

Eligible participants are between 21 and 60 years of age with a diagnosis of Alcohol Use Disorder according to DSM-5. Further, they need to be abstinent after detoxification for at least 5 days. All participants must have the ability to provide fully informed consent and to use self-rating scales. Hence, they also need to have a sufficient understanding of the German language.

Participants will be excluded if they have a lifetime history of DSM-5 bipolar or psychotic disorder, or substance dependence other than alcohol or nicotine dependence; a current threshold DSM-5 diagnosis of a current (hypo)manic episode, major depressive disorder, generalized anxiety disorder, posttraumatic stress disorder (PTSD), borderline personality disorder, or obsessive compulsive disorder. Other exclusion criteria include current substance use other than nicotine and/or mild to moderate recreational use of cannabis as evidenced by positive urine test; a history of severe head trauma or other severe central neurological disorders (e.g. dementia, Parkinson’s disease, multiple sclerosis); pregnancy or nursing infants; and use of medications or drugs known to interact with the Central Nervous System (CNS) within the last 10 days, with testing at least four half-lives post last intake.

### Power calculations

In order to conduct our effect size calculation two studies were used: Hartwell et al. [28], with an effect size of f = .59 in a study focusing on the anterior cingulate cortex in smokers and Kirsch et al. [39], which investigated heavy social drinkers with an effect size of f = .54 for VS cue-reactivity. Additionally, we examined a study by Tang, Tang, & Posner [50] with an effect size of f = .475 on activation in prefrontal control structures after a mindfulness-based intervention. Since the above-mentioned effect sizes originate from small samples, they might be an overestimation of real effect sizes [9]. Thus, we carefully assume an intermediate effect size of f = .3. We conducted the sample size estimation by means of the G*Power software tool version 3 [19]. The region of interest (VS, 200 voxels) was amended for multiple testing based on Bonferroni correction. As a result of this effect size, we require a sample size of 88 participants to attain a significant effect at *p* < .05 (Bonferroni corrected for multiple testing, region of interest mask of 500 voxels) with a power of 80%. With this sample size of 88 participants, we will be able to identify small effects of f = .15 for our primary clinical outcomes with a power of 95% (group by time interaction).

### Screening

For conduct of the screening, a trained clinical psychologist administers the Structured Clinical Interview for DSM-V (SKID-5-CV) after the participant gives informed consent. Further, participants are asked for a urine sample to apply a drug test and, if applicable, a pregnancy test.

### Baseline assessment

Following the group assignment, participants complete a baseline assessment that begins with a screening session, which determines whether the participants qualify for this study, and includes questionnaires, a neuropsychological assessment and an fMRI session. The questionnaires are focused on demographics, alcohol use, current medication and clinical symptoms. For a comprehensive list of the questionnaires used, please see Table 1. During the neuropsychological assessment, participants perform a dot probe task with alcohol stimuli to assess attentional bias for alcohol [53] and a dimensional card sorting task to measure executive functioning [22]. Then, participants are instructed to set three goals for the intervention. The fMRI session consists of a cue-reactivity paradigm to reflect incentive salience [47], a delay discounting task [21] investigating impulsivity [41] and a stop signal task to assess motor inhibition [23]. Participants who receive MBRP additionally receive an introduction to the program and a short exercise in mindfulness.

**Table 1:** Chart of measurements implemented in the study

**Measurement**	S01	MRI 1/baseline	MBRP	MRI 2	MRI 3	MRI 4	MRI5/ Post	FU1/2	FU3
Consent Form for the Study	X								
Sociodemographic Information		X							
Structured Clinical Interview (SKID – I)	X								
Drinking Assessment Interview (Form90)		X							
Urine Sample (Pregnancy & Drugs)	X								
Breath Alcohol Test									X
Current Medication	X								
Current Drug Use	X							X	X
MBRP Confidence Scale				X	X	X			X
MBRP Fidelity Scale			X						
Clinical & Personality Questionnaires	S01	MRI 1/ baseline		MRI 2	MRI 3	MRI 4	MRI5/ Post	FU1/2	FU3
General Depression Scale (Allgemeine Depressionsskala)		X							
Positive and Negative Affect Scale (PANAS)		X							
Perceived Stress Scale (PSS)		X							
Behavioral Inhibition/Approach System (BIS/BAS)		X							
Baratt Impulsiveness Scale (BIS-15)		X							
Fagerstrom Test of Nicotine Dependence (FTND)		X							
Goal Attainment Scaling		X							X
Sensory Inventory		X							
Alcohol-Related Questionnaires	S01	MRI 1/ Baseline		MRI 2	MRI 3	MRI 4	MRI5/ Post	FU1/2	FU3
Quick Drinking Assessment Interview (Form 90)								X	X
Alcohol Dependence Scale (ADS)		X							
Alcohol Abstinence Self-Efficacy Scale (AASE)		X							X
German Inventory of Drinking Situations (DITS-40)		X							
Obsessive Compulsive Drinking Scale (OCDS)		X							X
Alcohol Urge Questionnaire (AUQ)		X							X
Craving-Automatized-Scale-Alcohol (CASA)		X							X
Neuropsychological Tests	S01	MRI 1/ Baseline		MRI 2	MRI 3	MRI 4	MRI5/ Post	FU1/2	FU3
Vocabulary test		X							
Dot Probe Task with Alcohol stimuli		X							
Dimensional Card Sorting Task		X							
MRI	S01	MRI 1/ Baseline		MRI 2	MRI 3	MRI 4	MRI5/Post	FU1/2	FU3
Anatomical image (MPRAGE)		X		X	X	X	X		
Stop Signal Task (SST)		X					X		
Cue-Reactivity Paradigm		X					X		
Delay Discounting		X					X		
Resting State		X		X	X	X	X		
Neurofeedback				X	X	X			
Transfer				X		X			
Craving (Visual Analog Scale) pre & post scanning				X	X	X			
Perceived control over NFB (Visual Analog Scale)				X	X	X			

S01: Screening assessment and Baseline. MRI 1: MRI scanning appointment during baseline. MRI 2–4: rtfMRI NFB scanning appointments. MRI 5: MRI scanning appointment after the rtfMRI NFB sessions. FU 1–2: monthly follow-ups via telephone. FU 3: catamnestic interview (face-to-face)

### MBRP description

The MBRP training consists of five sessions, which are based on the Mindfulness-Based Relapse Prevention for Addictive Behaviors by [7]. The first session begins with a Raisin Meditation followed by a hand-out with a definition of mindfulness and explains two key concepts of mindfulness: the first one being the instruction not to judge and the second concept being the introduction of mindfulness as an alternative strategy to the mode of the autopilot. Acting on autopilot means experiencing the automatic behavior we experience during everyday life, which describes a condition of acting without conscious self-control or awareness. By stopping and observing, participants are sensitized to their thoughts and feelings. At the end of the first session the patients receive homework in form of a sheet of paper, in which they are instructed to write down time, date, feelings thoughts and behavioral impulses whenever they feel craving. The next session begins with a Body Scan Meditation. Then, difficulties during meditation are discussed and the participants are handed a worksheet which explains the concept of acceptance. The third session starts with Mindful Breathing Meditation. Furthermore, risk situations for relapse are examined and the SOBER Breathing Exercise is introduced. The participants are advised to practice the latter at least 5 minutes per day in order to be able to use this technique in risk situations which involve heavy craving. Upon a rehearsal of the SOBER Breathing training, the fourth session focuses on further examination of risk factors and a mindful way to deal with the latter. In the last session, which, as before, starts with SOBER Breathing, the homework sheet from the first session is examined. Participants receive a handout in which they are instructed to fill out answers relating to the importance of mindfulness. The session ends with a Completion Meditation. After the last session, participants who took part in the MBRP are asked to answer the Mindfulness-Based Relapse Prevention Adherence and Competence Scale [7].

### Neurofeedback setup

rtfMRI NFB is executed at a Siemens 3 T Scanner (Siemens Healthineers, Erlangen, Germany) at the Central Institute of Mental Health in Mannheim, Germany. rtfMRI NFB is delivered on three separate training days within 2 weeks. A T1-weighted anatomical MPRAGE scan, a functional resting state scan and three neurofeedback runs are performed during each session. For the EPIs we will use state-of-the-art multiband sequences.

To allow for neurofeedback signal extraction, the imaging computer transfers reconstructed DICOM images to a laptop which preprocesses the data and calculates the feedback signal using in-house scripts written in MATLAB (MathWorks Inc., Sherborn, MA, USA) based on SPM12 functions (Wellcome Department of Cognitive Neurology, London, UK).

**Fig. 2:**
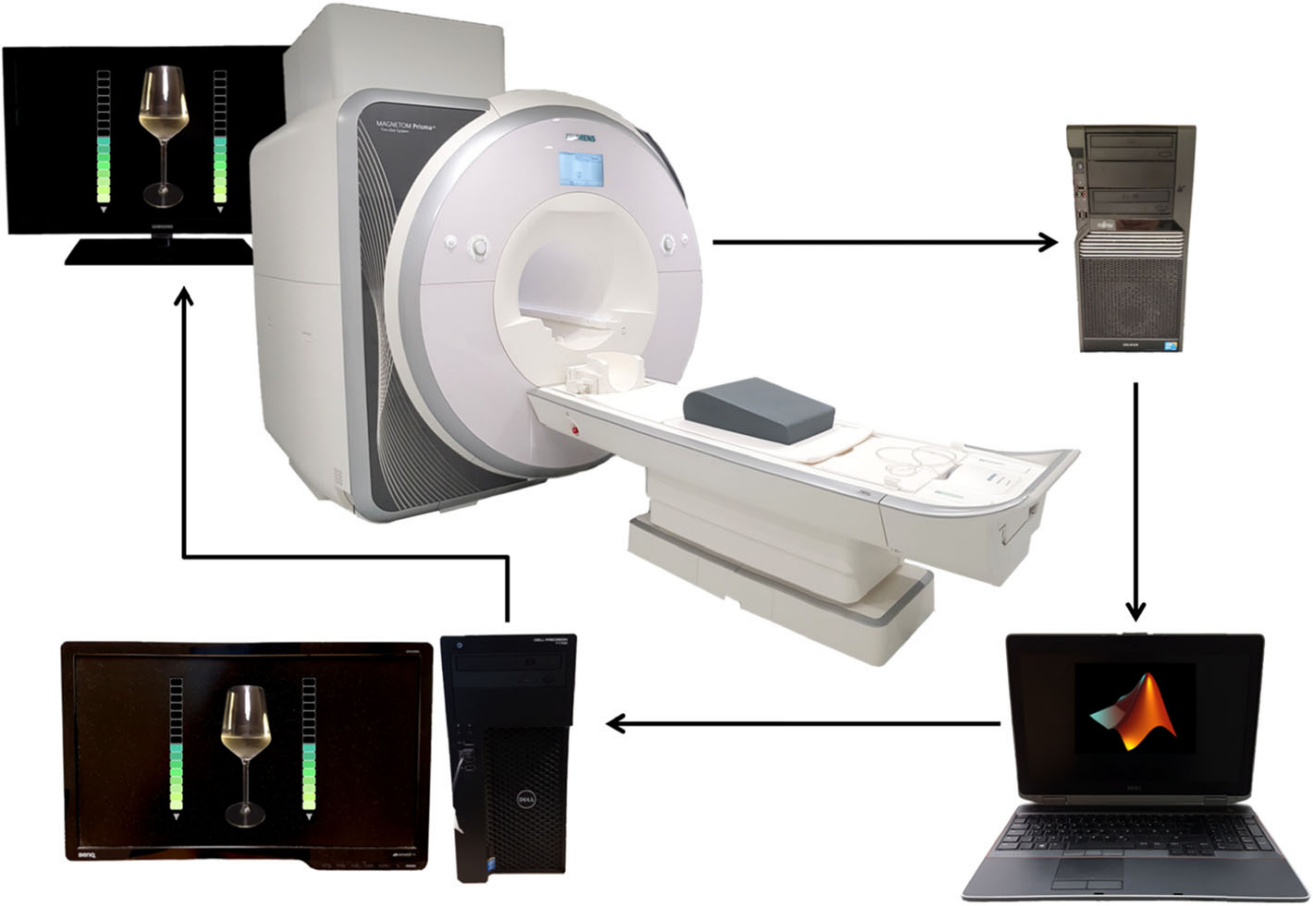
rtfMRI Neurofeedback Setup. The scanner computer reconstructs the obtained images and sends them to a laptop with in-house MATLAB scripts for preprocessing and extraction of the neurofeedback signal. The feedback value calculated is then forwarded to a computer with Presentation software and displayed as a thermometer value. The rights to this figure are reserved by the authors

At the start of each session, the obtained anatomical image undergoes segmentation and normalization to MNI space. For warping of the masks of the target regions into subject space, the inverse deformations of the normalization are used. During the rtfMRI NFB procedure, functional images are pre-processed with realignment and re-slicing. In a next step, the mask is adjusted to the latest image acquired and mean intensity values from the voxels in the target regions are derived. At each time step the whole available signal is corrected for nuisance effects. For display of the NFB signal, intensity values acquired in the presence of alcohol images are averaged over the last 3 volumes to allow for a stabilization of the feedback signal. Percent signal change in relation to the previous fixation cross is calculated and adapted to the maximum absolute signal change. The calculated signal is then sent to a presentation computer with Presentation software (Neurobehavioral Systems, Inc., Albana, CA, USA) where it is implemented as a thermometer value besides photos of the alcoholic drink of choice of the participant (beer, wine or schnapps) shown in randomized order (see Fig. 2). Update of the thermometer display takes place after every acquired volume.

### Task design

**Fig. 3:**
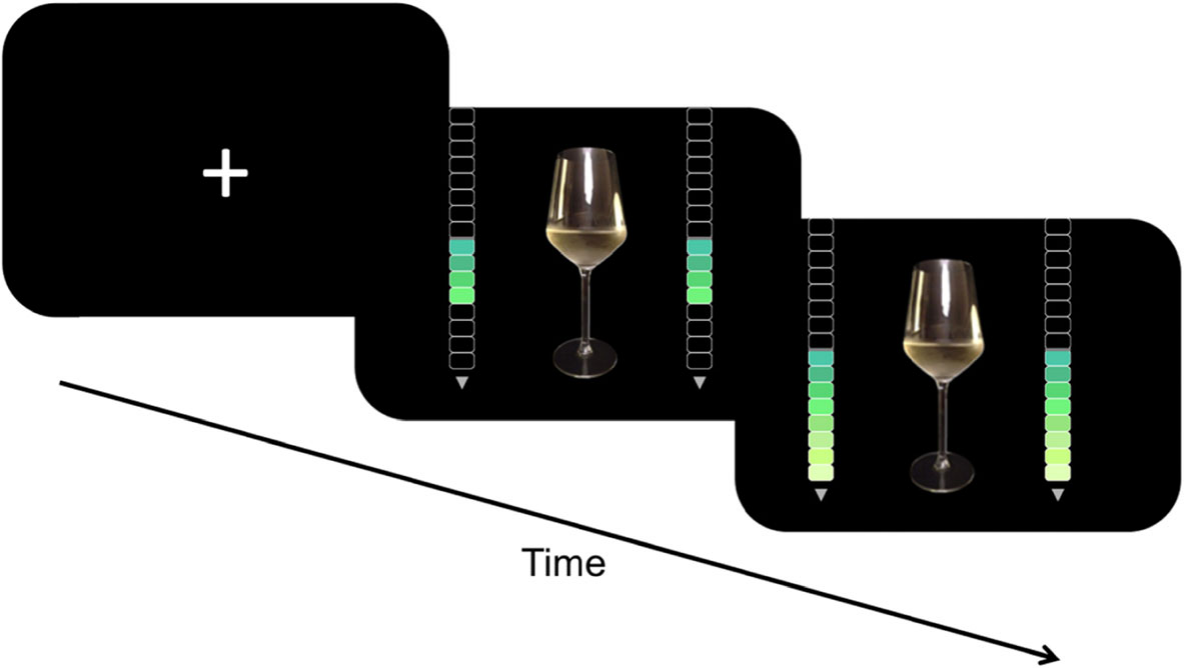
Experimental Design Neurofeedback. a) A fixation cross is presented to the participant in the scanner which is followed by an image of the preferred alcoholic beverage (beer or wine) alongside a thermometer to the left and right. The thermometer value displayed is updated every TR to represent the latest value. The rights to this figure are reserved by the authors

**Fig. 4:**
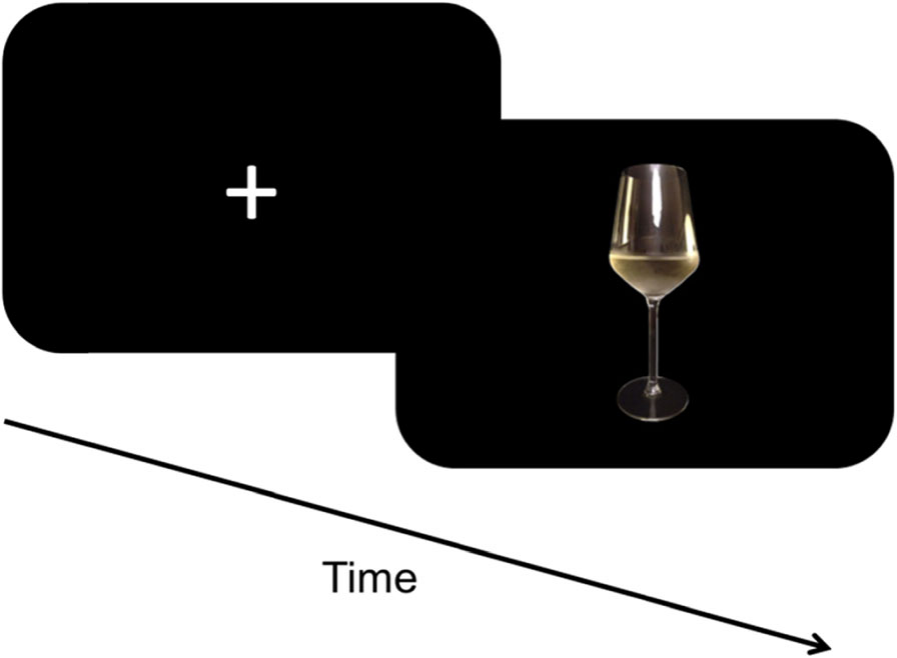
Experimental Design Transfer Run. During the transfer run no feedback is shown to the participant and the instruction is to apply the same strategies as in Fig. 3. The rights to this figure are reserved by the authors

The NFB sessions follow a block design in which a fixation cross and an image of an alcoholic beverage of choice are presented alternately. 10 s after the onset of the alcoholic beverages, a feedback thermometer appears on the screen which is updated every TR (Fig. 3). After two runs of NFB, a transfer run without the feedback component follows (Fig. 4), except for the second training session in which all runs are NFB runs.

A visual craving scale is used to evaluate the participant’s craving before and after the scanning. Additionally, participants fill in questionnaires (see Table 1).

Before the NFB starts, participants of the MBT group are reminded of their MBT training and are asked to apply the strategies they learned in the sessions. The TAU group receives sham instructions.

### Follow-up assessments

The follow-up assessments are conducted monthly, beginning after the last rtfMRI session. In total, participants are followed-up over a period of 3 months. During the first two follow-ups, participants are contacted via phone and asked whether they use any medication related to alcohol dependence. Additionally, they are inquired about their use of drugs and a possible alcohol relapse. During the last follow-up, which takes place at the CIMH 3 months after the last rtfMRI session, participants are requested to fill out alcohol-related questionnaires and are asked about substance use. For the catamnestic interview, participants are entitled to receive a monetary compensation.

### Statistical analyses

Offline analyses are conducted with SPM (Wellcome Department of Cognitive Neurology, London, UK) in MATLAB (MathWorks Inc., Sherborn, MA, USA) and include slice-time correction, realignment, segmentation of the anatomical image, normalization to MNI space and smoothing. In our first level analyses, we convolve the time courses with the canonical hemodynamic response function and add them to the General Linear Model (GLM) which also includes 6 movement parameters. The second level analyses comprise correlations with clinical variables and potentially predictive markers for the future course of the disease.

Missing data will be excluded from the analyses. Physiological activity (cardiac and respiratory) will also be recorded and then included in the first level to correct for noise of physiological nature. This will be performed via Global Signal Regression (GSR) and model-based physiology correction [52].

Clinically, the primary outcomes include the number of relapses and the number of days until relapse during the 3-months follow-up. From a neural perspective, the increase of the ability to voluntarily modulate brain activation to alcohol cues after training in the VS serves as a secondary outcome, whereas craving measures such as the Alcohol Abstinence Self-Efficacy Scale [18] will be analyzed as a primary outcome. Another secondary outcome is the influence of combined MBRP and rtfMRI NFB training on functional changes in brain networks of cognitive control (Stop Signal Task (SST), delay discounting).

### Data management and dissemination

All questionnaires are collected online with REDCap (Research Electronic Data Capture) software and are password-protected with a two-factor authentication. The study team is responsible for data management and monitoring of the data. Personal information is stored separately from the study data. All data are stored on local servers. We will present interim analyses of the data at national and international conferences and will publish the final results in peer-reviewed scientific reports. Authorship will be determined based on the recommendations for safeguarding good scientific practice by the German Research Foundation [17].

## Discussion

In this project we assess the efficacy of regulating cue-reactivity by means of rtfMRI NFB in AUD patients and investigate the impact of MBRP on the NFB procedure and the clinical outcome. To date, there is no other study that combines a mindfulness-based intervention specifically designed to prevent relapse in AUD with an innovative neuroscientific state-of-the-art treatment approach. The outcome will help to further understand the involvement of VS functioning in AUD and to develop novel interventions targeted at mechanisms of addiction. Additionally, our study will provide further insights on neurobiological changes in the brain as a consequence of rtfMRI NFB and MBRP interventions, which will be conducted either apart or combined.

The innovation of this study is that it will be the first of its kind to combine therapeutic and neurobiological interventions in order to treat alcohol use disorder. Another distinctive feature is the high degree of comparability between the various conditions that we assess. For example, we will be able to evaluate differences between participants receiving MBRP and TAU regarding NFB outcome. Furthermore, we can investigate changes in resting state networks before and after application of MBRP.

To date, the literature on rtfMRI NFB in AUD is relatively limited. An advantage of our study will be the homogeneity of the study population which enables a comparison of real and sham NFB within the same patient group instead of comparing this patient group to healthy controls [14, 35]. Importantly, this study also allows for investigations of neuropsychological measurements of the AUD patients and how they might be related to clinical features of NFB success which facilitates extensive insights into this disorder.

Design-wise, our rtfMRI NFB training composes three sessions, which is in line with studies showing that a training effect can only be found after two sessions, and that more than two sessions are required to detect clinical improvement [45, 49]. Clinical improvement is also expected to be seen in the follow-up [45] which in our study will be conducted after 3 months. From a technical standpoint, we are capable of implementing the state-of-the-art methods that fMRI can offer by using multiband sequences [55].

This protocol presents with some drawbacks likewise. First, it is known that AUD patients presenting with habit-like addiction behavior are more likely to demonstrate a change in striatal activity from ventral to dorsal parts of the striatum [51]. Hence, the dorsal striatum would also represent a suitable target to use. However, we chose the VS here because we want to target reward dependent cue-reactivity especially reached by our alcohol pictures and not a general habit process.

Furthermore, it is also arguable whether one should use the same paradigm in male and female participants. A previous study has shown that sex differences in cue-reactivity exist [31] which are manifested by significant increases in activity of the VS as a consequence of a cue-reactivity alcohol task in males but not in females. In this study we will still continue assessing both sexes in order to include sex differences in our analysis, but likely our study will include a higher ratio of male participants due to the basic rate in our patient population.

Another point to discuss is that rtfMRI NFB can only be performed successfully by a proportion of people, but likely not by everyone. In a recent review paper Fede, Dean, Manuweera, & Momenan [20] summarized findings from a multitude of rtfMRI NFB studies and mention that only 50–75% of subjects are capable of changing brain activity into the desired direction.

Importantly, confidence of the subjects in mastering the task is related to successful rtfMRI NFB performance [34]. There are also other options regarding NFB design, e.g. target definition could be done by means of a functional localizer or a cue exposure task [14, 35] instead of using a predefined mask, but we consider the mask approach the most suited one because we want to ensure that we target the exact same area in all subjects. Regarding the sham feedback, we chose to feed back the signal of a different ROI (auditory cortex) in the same patient and we abstained from showing the feedback value of a different participant. For this reason, we decided to keep the “deception” of the participants as low as possible. In the very unlikely event of side effects reported by the participants, the principal investigators will determine continuation or termination of the study or subparts of it.

In sum, the study will collect significant data on the efficacy of adding mindfulness-based training to rtfMRI NFB in AUD. In addition, the extensive amount of imaging data will allow us to draw meaningful conclusions on the NFB method and to determine potential clinical predictors.

The study is pre-registered at clinicaltrials.gov (NCT04366505).

## Data Availability

Due to protection of sensitive personal data it is not allowed to release raw fMRI or clinical data. However, after finalization of the study we will publish summary data in a repository.

## Abbreviations

AUD: Alcohol Use Disorder
CIMH: Central Institute of Mental Health Mannheim
CNS: Central nervous system
dlPFC: Dorsolateral prefrontal cortex
DSM – 5: Diagnostic and Statistical Manual of Mental Disorders (5th Edition)
e.g.: Exempli gratia
EPI: Echo-planar imaging
fMRI: Functional magnetic resonance imaging
GLM: General Linear Model
GSR: Global Signal Regression
MBRP: Mindfulness Based Relapse Prevention
MBT: Mindfulness-based treatment
MRI: Magnetic resonance imaging
NFB: Neurofeedback
PTSD: Posttraumatic Stress Disorder
REDCap: Research Electronic Data Capture
rtfMRI NFB: Real time functional magnetic resonance imaging neurofeedback
SKID – 5 – CV: Structural Clinical Interview for DSM – 5
SST: Stop signal task
TAU: Treatment as usual
TE: Echo time
TR: Time of repetition
vmPFC: Ventromedial prefrontal cortex
VS: Ventral striatum
